# The Role of YouTube in Patient Education on Renal Oncocytoma: A Quality Assessment

**DOI:** 10.7759/cureus.98880

**Published:** 2025-12-10

**Authors:** Mohammed Saad, Mujahid Abuagla Dafalla Hamed

**Affiliations:** 1 Urology, Barts Health NHS Trust, London, GBR; 2 Faculty of Medicine, Al Neelain University, Khartoum, SDN

**Keywords:** benign renal mass, renal neoplasm, renal oncocytoma, youtube study, youtube videos

## Abstract

Introduction

The increasing use of advanced imaging techniques has led to more frequent detection of renal masses, including benign tumours like renal oncocytomas. Although typically asymptomatic, these tumours can complicate diagnosis due to their resemblance to renal cell carcinoma (RCC) on imaging studies. Treatment strategies for renal oncocytomas depend on tumour size, symptoms, and imaging features. As patients increasingly turn to online platforms for health information, YouTube has become a popular source for educational videos. However, the quality and reliability of such videos vary. This study evaluates YouTube videos on renal oncocytoma to assess their role in patient education.

Materials and methods

A systematic search of YouTube was conducted on September 5, 2025, using the keyword "renal oncocytoma." A total of 60 videos were screened, and 10 met the inclusion criteria. The educational quality and reliability of the selected videos were assessed using the Global Quality Score (GQS) and the DISCERN instrument. Video popularity was assessed with the Video Power Index (VPI). A one-sample t-test was used to compare the average GQS and DISCERN scores with predefined benchmarks. Spearman’s rank correlation coefficient (ρ) was used to evaluate associations between these metrics.

Results

The videos analysed had a mean GQS of 3.7 ± 0.90, significantly higher than the midpoint score of 3 (p = 0.037), indicating moderate educational quality. DISCERN scores revealed that while the videos clearly stated their aims (mean score 4.8 ± 0.98, p = 0.0003), they often fell short in providing detailed, patient-centered information. The mean score for describing treatment options was 2.1 ± 1.81 (p = 0.15), and there was a notable lack of transparency regarding information sources, with a mean score of 1.7 ± 1.23 (p = 0.008). Although the videos provided current information (mean score 4.4 ± 0.94, p = 0.001), they did not adequately address uncertainties (mean score 2.0 ± 1.00, p = 0.011) or support shared decision-making (mean score 2.3 ± 1.65, p = 0.207). The VPI revealed no significant correlation with the quality metrics, with a moderate negative correlation between GQS and VPI (ρ = -0.424, p = 0.299) and a weak negative correlation between DISCERN and VPI (ρ = -0.141, p = 0.757).

Conclusion

This study highlights the potential of YouTube as an educational tool for renal oncocytoma, but it also underscores significant gaps in the quality and reliability of the information provided. The videos often lack comprehensive discussions on treatment options, transparency, and addressing uncertainties, which are essential for informed patient decision-making. Although YouTube can serve as a starting point for general information, patients should consult healthcare professionals for accurate, personalised advice. Efforts to improve the quality of health-related content on YouTube are needed to better support patient education.

## Introduction

The widespread adoption of advanced imaging technologies has led to an increased detection of renal masses, facilitating the identification of both benign and malignant tumours. Among benign renal masses, renal oncocytomas are relatively common, originating from the proximal renal tubules [1,2]. These tumours are frequently asymptomatic and are often discovered incidentally during imaging studies performed for unrelated reasons. However, in some cases, renal oncocytomas may present with symptoms such as flank pain or haematuria [1].

On imaging, renal oncocytomas frequently exhibit characteristics similar to those of renal cell carcinoma (RCC), including well-defined, enhancing masses on CT or MRI scans. This overlap in imaging features can complicate the differentiation between the two, often necessitating a biopsy for definitive diagnosis [1,3].

Although renal oncocytomas are typically small in size, larger tumours have also been reported, which may complicate both diagnosis and treatment decisions [4-6]. In rare instances, metastasis from renal oncocytomas has been observed [7,8].

Management of renal oncocytomas is influenced by tumour size, symptoms, and imaging characteristics. For small, asymptomatic tumours, conservative management with active surveillance, including regular imaging (such as ultrasound, CT, or MRI) to monitor tumour growth, is often appropriate. However, surgical intervention, such as partial nephrectomy, may be required for larger or symptomatic tumours, or when there is uncertainty regarding the diagnosis and malignancy cannot be excluded [8-10]. In certain cases, minimally invasive treatments may provide alternative options to surgery [11].

With the increasing prevalence of digital media, more patients are turning to online sources for health-related information, with platforms like YouTube becoming increasingly popular. Since its inception over two decades ago, YouTube has offered free access to a wide range of videos, including content focused on medical education, procedural demonstrations, and patient guidance [12]. When videos are well-structured and evidence-based, the platform has the potential to enhance health literacy and serve as a valuable complement to traditional patient education methods [13,14].

However, the quality and reliability of medical content on YouTube can vary significantly. Several studies have highlighted that a considerable proportion of health-related videos contain incomplete, misleading, or inaccurate information, even when uploaded by healthcare professionals [15,16]. Such inaccuracies or poorly explained content may contribute to misinformed health decisions, increased anxiety, or inappropriate self-management.

The current study aims to evaluate YouTube videos on renal oncocytoma and investigate how online video content may influence patient understanding and decision-making related to the condition.

## Materials and methods

On September 5, 2025, a systematic search of YouTube [24] was conducted using the keyword “renal oncocytoma.” The first 60 videos retrieved through the default relevance sorting were screened for eligibility. Videos were included if they were in English, had a duration of no more than 25 minutes, and focused specifically on renal oncocytoma. Videos were excluded if they were intended for medical professionals, duplicates, less than 1 minute or primarily focused on histology instruction.

After applying these criteria, 10 videos met the inclusion requirements and were selected for analysis. The following characteristics were recorded for each video: upload date, duration, number of views, likes, and dislikes.

The overall educational quality of each video was evaluated using the Global Quality Score (GQS), a validated five-point Likert scale, where 1 = poor, 2 = generally poor, 3 = moderate, 4 = good, and 5 = excellent [17].

The educational reliability of each video was assessed using the 16-item DISCERN instrument (total score range: 16-80), which categorises videos as excellent (>63), good (51-62), fair (39-50), poor (27-38), or very poor (<27) [18-20].

The popularity of each video was evaluated using the Video Power Index (VPI), calculated by dividing the number of likes by the total of likes and dislikes, then multiplying the result by 100. This metric reflects the video's popularity by comparing the proportion of positive feedback to overall viewer engagement [21-23].

A standardised evaluation questionnaire (Table 1) was developed to ensure consistency in the assessment process. Two evaluators independently reviewed all videos, and any discrepancies in scoring were addressed through consensus discussion.

**Table 1:** The questionnaire developed for assessing the videos

General Information	Upload Date
General Information	Duration
General Information	Views
General Information	Likes
General Information	Dislikes
GQS	Global Quality Score (1–5)
VPI	Video Power Index
DISCERN 1	Are the aims of the video clear?
DISCERN 2	Does it achieve its aims?
DISCERN 3	Is it relevant to patients?
DISCERN 4	Are sources of information clearly stated?
DISCERN 5	Is the information balanced and unbiased?
DISCERN 6	Are additional sources listed?
DISCERN 7	Is the video current and up to date?
DISCERN 8	Does it describe how each treatment works?
DISCERN 9	Does it describe the benefits of each treatment?
DISCERN 10	Does it describe the risks of each treatment?
DISCERN 11	Does it describe what would happen without treatment?
DISCERN 12	Does it describe how choices affect quality of life?
DISCERN 13	Is it clear more than one treatment option exists?
DISCERN 14	Does it support shared decision-making?
DISCERN 15	Does it mention areas of uncertainty?
DISCERN 16	Overall quality rating
Overall DISCERN score	

A one-sample t-test was performed to compare the average Global Quality Score (GQS) for these videos against the midpoint score of 3, which indicates a video of moderate educational quality. We also conducted a one-sample t-test to determine whether the average DISCERN score for these videos significantly differed from the threshold score of 51, which is considered the cutoff for "good" reliability. Additionally, continuous variables were summarised as mean ± standard deviation. Spearman’s rank correlation coefficient (ρ) was used to evaluate associations between DISCERN and VPI, GQS and VPI, as well as DISCERN and GQS. A p-value of < 0.05 was considered statistically significant. All analyses were conducted using GraphPad Prism 10 software.

## Results

A total of 10 videos met the inclusion criteria and were selected for analysis. The upload dates of the videos ranged from 2020 to 2025, with a mean of 2.625 ± 1.33 years. The durations of the videos ranged from 1:28 to 22:27 minutes, with a mean duration of 7.31 ± 6.21 minutes. The number of views ranged from 13 to 82,000, with a mean of 12,258 ± 27,373 views.

The mean Global Quality Score (GQS) for the videos was 3.7 ± 0.90, with a t-statistic of 2.46 and a p-value of 0.037, indicating a statistically significant deviation from the midpoint score of 3.

**Figure 1:**
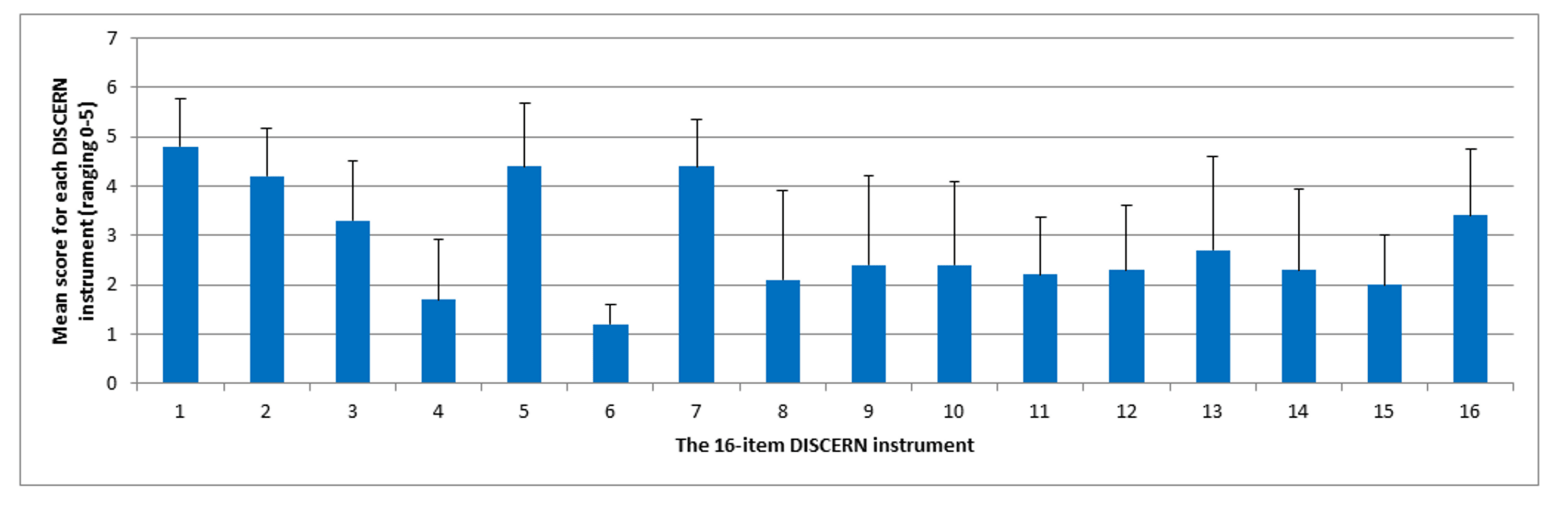
The 16-item DISCERN instrument evaluation for the videos

The DISCERN evaluation revealed varying levels of quality across different criteria (Table 2, Figure 1). The mean score for clarity of aims was 4.8 ± 0.98, with a p-value of 0.0003, suggesting that the videos clearly articulated their aims. The mean score for achievement of aims was 4.2 ± 0.98, with a p-value of 0.004, indicating that the videos effectively met their stated objectives. The relevance of the videos to patients had a mean score of 3.3 ± 1.22, with a p-value of 0.45, indicating no statistically significant difference from the midpoint score.

**Table 2:** Presents the mean scores for each DISCERN criterion, summarising the evaluation results.

		Mean	SD	t-statistic	p-value
DISCERN 1	Are the aims of the video clear?	4.8	0.98	5.81	0.0003
DISCERN 2	Does it achieve its aims?	4.2	0.98	3.87	0.004
DISCERN 3	Is it relevant to patients?	3.3	1.22	0.78	0.45
DISCERN 4	Are sources of information clearly stated?	1.7	1.23	-3.34	0.008
DISCERN 5	Is the information balanced and unbiased?	4.4	1.29	3.42	0.007
DISCERN 6	Are additional sources listed?	1.2	0.4	-14.29	0.0001
DISCERN 7	Is the video current and up to date?	4.4	0.94	4.71	0.001
DISCERN 8	Does it describe how each treatment works?	2.1	1.81	-1.57	0.15
DISCERN 9	Does it describe the benefits of each treatment?	2.4	1.81	-1.05	0.32
DISCERN 10	Does it describe the risks of each treatment?	2.4	1.68	-1.13	0.29
DISCERN 11	Does it describe what would happen without treatment?	2.2	1.17	-2.16	0.062
DISCERN 12	Does it describe how choices affect quality of life?	2.3	1.31	-1.69	0.13
DISCERN 13	Is it clear more than one treatment option exists?	2.7	1.90	-0.50	0.625
DISCERN 14	Does it support shared decision-making?	2.3	1.65	-1.34	0.207
DISCERN 15	Does it mention areas of uncertainty?	2	1	-3.16	0.011
DISCERN 16	Overall quality rating	3.4	1.35	0.94	0.373

Regarding the clarity of sources, the mean score was 1.7 ± 1.23, with a p-value of 0.008, suggesting that the videos did not clearly state their sources. The balance and bias of the information had a mean score of 4.4 ± 1.29, with a p-value of 0.007, indicating that the videos provided balanced and unbiased information. The mean score for listing additional sources was 1.2 ± 0.4, with a p-value of 0.0001, highlighting that additional sources were rarely included in the videos.

The currency of the videos was evaluated with a mean score of 4.4 ± 0.94 and a p-value of 0.001, indicating that the videos were generally up to date.

For the criterion describing treatments, the mean score was 2.1 ± 1.81, with a p-value of 0.15, suggesting that, on average, the videos did not effectively describe how each treatment works, but this difference was not statistically significant. Similarly, the mean score for describing the benefits of each treatment was 2.4 ± 1.81, with a p-value of 0.32, and the risks of each treatment had a mean score of 2.4 ± 1.68, with a p-value of 0.29, indicating that the videos did not effectively describe these aspects, but the differences were not statistically significant.

The videos also did not effectively describe what would happen without treatment, with a mean score of 2.2 ± 1.17 and a p-value of 0.062, although this difference was not statistically significant. The mean score for evaluating how choices affect quality of life was 2.3 ± 1.31, with a p-value of 0.13. The videos did not clearly highlight multiple treatment options, with a mean score of 2.7 ± 1.90 and a p-value of 0.625. The mean score for supporting shared decision-making was 2.3 ± 1.65, with a p-value of 0.207, indicating that the videos were not significantly supportive of shared decision-making.

The mention of areas of uncertainty was rated with a mean score of 2.0 ± 1.0 and a p-value of 0.011, indicating a statistically significant lack of mention of uncertainties in the videos. The overall quality rating of the videos had a mean score of 3.4 ± 1.35, with a p-value of 0.373, suggesting that the overall quality did not significantly differ from the midpoint score.

The overall DISCERN score for the videos had a mean of 45.7 ± 17.54, with a p-value of 0.372, indicating no significant difference from the expected quality level for the videos.

Spearman’s rank correlation coefficient (ρ) was calculated to evaluate the associations between DISCERN Scores, Global Quality Scores (GQS), and Video Power Index (VPI) (Table 3). A moderate positive correlation was observed between DISCERN scores and GQS, suggesting that videos with higher educational quality tended to have higher reliability as measured by the DISCERN instrument. However, this correlation did not reach statistical significance. A weak negative correlation was found between DISCERN scores and VPI, indicating a minimal association between video reliability and viewer engagement. Additionally, a moderate negative correlation was observed between GQS and VPI, suggesting that videos with higher educational quality may attract lower levels of viewer engagement. None of the associations between the variables were statistically significant (all p-values > 0.05), indicating that while trends were noted, the relationships between video quality and engagement did not reach statistical significance in this dataset.

**Table 3:** Spearman’s rank correlation coefficient (ρ) evaluation between DISCERN Scores, Global Quality Scores (GQS), and Video Power Index (VPI)

Variables compared	Spearman’s ρ	p-value
DISCERN Score vs GQS	0.655	0.087
DISCERN Score vs VPI	-0.141	0.757
GQS vs VPI	-0.424	0.299

## Discussion

The findings of this study provide important insights into the educational quality and reliability of YouTube videos related to renal oncocytoma. The results indicate that while the overall quality of the videos was moderate to good, gaps exist in terms of providing comprehensive, reliable, and patient-centered information. With an average Global Quality Score (GQS) of 3.7, the videos on renal oncocytoma were generally able to provide useful overviews of the condition, suggesting they could be valuable resources for patients seeking initial information about the disease. However, the variability in quality and content highlights the need for careful consideration when using such videos as educational tools [13,16].

A key finding is the strong clarity of the videos’ aims, with a mean score of 4.8 for clarity and 4.2 for achieving those aims. This suggests that most videos were clear about their objectives and were successful in communicating the basic aspects of renal oncocytoma. Despite this, there was a noticeable weakness in explaining critical aspects such as treatment options, associated risks and benefits, and the overall impact of treatment decisions on quality of life. These topics, which are crucial for patient decision-making, were not adequately addressed, with mean scores for treatment descriptions hovering around 2.1 to 2.4. Furthermore, the videos failed to present a clear discussion of alternative treatment options and did not encourage shared decision-making, a critical component of patient-centered care [12,18].

Another result was the lack of transparency regarding sources of information, with a mean score of 1.7 for the clarity of sources and 1.2 for the listing of additional sources. This lack of proper citation and reference is troubling, as patients might not be able to distinguish between evidence-based content and unreliable or unverified information. The absence of sources could undermine the credibility of the videos and may lead patients to make decisions based on incomplete or misleading information. Furthermore, the significant deficiency in addressing uncertainties around renal oncocytoma, as reflected by a mean score of 2.0, is another indication that these videos do not adequately inform patients about the areas where medical knowledge is still evolving or where the diagnosis and treatment decisions may involve some degree of uncertainty [15,19,21].

The videos generally offered current information, as reflected by a high score of 4.4 for currency. This suggests that the videos were up to date with the latest developments in renal oncocytoma, which is critical for ensuring that patients receive accurate and timely information. However, the lack of in-depth discussion about treatment options and their respective risks and benefits indicates that these videos may not be sufficient for patients looking for comprehensive guidance on managing their condition [8,9].

The correlation analyses showed that while there was a moderate positive correlation between the GQS and DISCERN scores, indicating that higher quality videos tended to be more reliable, these associations were not statistically significant. This suggests that high-quality educational content does not always equate to high reliability, and popular videos, as indicated by the Video Power Index (VPI), may not necessarily provide the most accurate or useful information for patients [22,23].

This study has several limitations. The sample size was relatively small, with only 10 videos meeting the inclusion criteria, which may limit the generalisability of the findings. YouTube content is dynamic and constantly changing, and videos may be added, removed, or updated over time. Therefore, the findings reflect only the state of the platform at the time the data were collected. The search strategy relied on a single keyword, which may have excluded relevant videos that used alternative terminology. By restricting the analysis to English content, the study may have missed educational resources presented in other languages. While validated tools like GQS and DISCERN were employed, the evaluation process still involved an element of subjective judgment, as it was conducted by only two reviewers. Lastly, viewer-related factors, including health literacy, prior knowledge, and personal interpretation, were not assessed, which may impact how patients perceive and derive benefit from the online video content.

## Conclusions

This study highlights YouTube's potential as an educational tool for patients seeking information on renal oncocytoma, offering moderate educational value and clear objectives. Weaknesses were found in areas such as transparency, treatment discussions and addressing uncertainties, which are essential for informed decision-making. While YouTube videos can provide useful introductory knowledge, they should be viewed as supplementary resources, not as substitutes for professional medical advice. Patients are encouraged to consult healthcare providers for personalised guidance, particularly regarding treatment options and risks. The study emphasises the need to improve the quality and reliability of health-related content on YouTube, with accountability shared among content creators, viewers, and healthcare professionals to ensure access to accurate evidence-based information.
